# Efficiency in PrEP Delivery: Estimating the Annual Costs of Oral PrEP in Zimbabwe

**DOI:** 10.1007/s10461-021-03367-w

**Published:** 2021-08-27

**Authors:** Collin Mangenah, Definate Nhamo, Stephano Gudukeya, Emily Gwavava, Chiedza Gavi, Progress Chiwawa, Sandra Chidawanyika, Polite Muleya, Noah Taruberekera, Ngonidzashe Madidi, Gertrude Ncube, Hilda Bara, Sue Napierala, Megan Dunbar, Theresa Hoke, Katharine Kripke, Frances M. Cowan, Kristine Torjesen, Fern Terris-Prestholt

**Affiliations:** 1 Centre for Sexual Health, HIV/AIDS Research, 4 Bath Road, Belgravia, Harare, Zimbabwe; 2 Pangaea Zimbabwe AIDS Trust (PZAT), Harare, Zimbabwe; 3 Population Services International Zimbabwe, Harare, Zimbabwe; 4 Ministry of Health and Child Care, Harare, Zimbabwe; 5 Harare City Health Department, Harare, Zimbabwe; 6 RTI International, Durham, NC USA; 7 FHI 360, Durham, NC USA; 8 Avenir Health, Takoma Park, MD USA; 9 International Public Health Department, Liverpool School of Tropical Medicine, Liverpool, UK; 10 London School of Hygiene and Tropical Medicine, London, UK

**Keywords:** Costs analysis, HIV, Oral PrEP, Continuation, Zimbabwe

## Abstract

Although oral PrEP is highly effective at preventing HIV acquisition, optimizing continuation among beneficiaries is challenging in many settings. We estimated the costs of delivering oral PrEP to populations at risk of HIV in seven clinics in Zimbabwe. Full annual economic costs of oral PrEP initiations and continuation visits were estimated from the providers’ perspective for a six-clinic NGO network and one government SGBV clinic in Zimbabwe (January–December 2018). Disaggregating costs of full initiation and incremental follow-up visits enabled modeling of the impact of duration of continuation on the cost per person-year ($pPY) on PrEP. 4677 people initiated oral PrEP, averaging 2.7 follow-up visits per person. Average cost per person initiated was $238 ($183–$302 across the NGO clinics; $86 in the government facility). The full cost per initiation visit, including central and direct costs, was $178, and the incremental cost per follow-up visit, capturing only additional resources used directly in the follow up visits, was $22. The average duration of continuation was 3.0 months, generating an average $pPY of $943, ranging from $839 among adolescent girls and young women to $1219 in men. Oral PrEP delivery costs varied substantially by scale of initiations and by duration of continuation and type of clinic. Extending the average oral PrEP continuation from 2.7 to 5 visits (about 6 months) would greatly improve service efficiency, cutting the $pPY by more than half.

**Supplementary Information:**

The online version contains supplementary material available at 10.1007/s10461-021-03367-w.

## Introduction

Sub-Saharan Africa (SSA) is home to nearly 61% of people living with HIV (PLHIV) globally [1]. Zimbabwe is among the countries worst affected, with an adult HIV prevalence of 14.7% and 1.2 million PLHIV [2]. In 2019 alone, 20,000 AIDS-related deaths and 40,000 new HIV infections were recorded in Zimbabwe [1]. Existing HIV prevention approaches, though often widely available at no cost in resource-limited settings, have been either characterized by low uptake in the case of voluntary medical male circumcision (VMMC) or inconsistently used in the case of male and female condoms [3–6]. Evidence from clinical trials showing effectiveness at preventing HIV acquisition of up to 96% when oral pre-exposure prophylaxis (PrEP) is combined with appropriate clinical monitoring led to the WHO guidelines recommending tenofovir-based daily oral PrEP for those at substantial risk of HIV acquisition [7, 8]. PrEP has also proven highly effective as a “bridge” to sustained antiretroviral therapy (ART) use for serodiscordant couples, with the HIV-seronegative partner taking the drug for a limited time when the HIV-seropositive partner is on ART but not yet virally suppressed or is not yet on ART [9–12].

In line with WHO guidance [13, 14], some countries with high HIV burdens introduced oral PrEP as part of their HIV prevention services. In 2016, Zimbabwe’s Ministry of Health and Child Care (MoHCC) launched the *Consolidated Guidelines for Antiretroviral Therapy for the Prevention and Treatment of HIV in Zimbabwe* [15]. These guidelines recommend oral PrEP to all individuals who are not living with HIV and perceive themselves to be at relatively high risk of HIV infection. Potentially eligible populations include adolescent girls and young women (AGYW), pregnant women in relationships with men of unknown status, HIV-seronegative partners in serodiscordant relationships, female and male sex workers (SWs), high-risk men who have sex with men (MSM), prisoners, long-distance truck drivers and transgender women.

For Zimbabwe, oral PrEP was initially introduced as part of the PEPFAR-funded Determined, Resilient, Empowered, AIDS-free, Mentored and Safe (DREAMS) initiative that aims to reduce HIV incidence among AGYW. PrEP was subsequently expanded to key populations (KPs), particularly MSM, transgender people, female SWs, serodiscordant couples, young women who sell sex and married people at substantial risk of HIV [16]. As oral PrEP delivery is scaled up across the country, an important consideration for MoHCC policy makers, funders and program implementers is how to optimize coverage and adherence among high-risk populations to maximize population‐level impact [17]. However, a dearth of program and unit cost data are available to facilitate optimization decisions both within and outside Zimbabwe, because few studies have attempted to estimate the initiation and continuation costs of PrEP delivery [17–19]. To our knowledge, no published study has estimated the costs of PrEP delivery in Zimbabwe to date.

To help bridge this evidence gap, we conducted a costing study to estimate the annual total program and unit costs of initiating and supporting continuation of clients on oral PrEP. This costing study was conducted in six social franchise clinics run by a non-governmental organization (NGO), Population Services International (PSI) Zimbabwe, offering integrated HIV and sexual and reproductive health (SRH) services across five provinces and in one government sexual and gender-based violence (SGBV) clinic﻿ in Zimbabwe. The primary costing objectives were to provide input into cost-effectiveness modeling and data for assessing resource needs associated with scaling up PrEP delivery. We also modeled the variation in cost per person-year on PrEP ($pPY) based on duration of continuation.

## Methods

### Study Setting

In 2017, PSI began providing oral PrEP as part of the package of SRH services offered to the general population, including key populations, in its stand-alone *New Start Centre (NSC)* clinics, which have been in operation since 1999 [20]. These clinics include the DREAMS-supported PSI sites in Bulawayo, Gweru, Mutare and Chipinge and the non-DREAMS sites in Harare and Masvingo [16]. In addition, a government 24–h SGBV clinic in Harare has offered oral PrEP integrated in existing services for KPs since June 2017 [21]. The MoHCC-approved regimen is a daily fixed-dose combination of 300 mg tenofovir disoproxil fumarate (TDF) and 200 mg emtricitabine (FTC) or lamivudine (3TC).

The costing study was undertaken in the six PSI clinics and the government SGBV clinic, where PrEP services are offered for free alongside other SRH and HIV prevention and treatment services implemented by private non-profit providers supported by PEPFAR. The 7 clinic sites were selected on the basis that they were the only ones in Zimbabwe that met the inclusion criterion of having delivered PrEP for at least 12 months to allow a full year’s costing. Sites were either stand alone or co-located with other clinics and were located in regions across the country in both small towns and large urban areas. Table 1 shows the characteristics of the sites which include the PSI-run sites (columns 1–4, 6), a clinic that PSI subcontracted to an implementing partner (column 5) and the government SGBV site (column 7). Further details of the services provided at the sites can be found in supplemental Table A1.

**Table 1:** Site characteristics for 7 Zimbabwe sites offering PrEP (2018)

Site	Site 1	Site 2	Site 3	Site 4	Site 5	Site 6	Site 7
Management	NGO site	NGO site	NGO site	NGO site	NGO partner run site	NGO site	Government SGBV site
Urban type	Large city	Medium size city	Medium size city	Large city	Medium size city	Small town	Large city
Site location	City stand-alone	City health clinic	City stand-alone	City stand-alone	City stand-alone	City stand-alone	City health clinic
Clinic size by total client visits (January–December 2018)	124,124	5070	22,356	53,214	28,217	3614	63,928
PrEP program start date	Nov 2016	Feb 2017	Nov 2016	Aug 2016	Aug 2016	Nov 2016	Jun 2017
Population served by clinic	General including KPs	General including KPs	General including KPs	General including KPs	General including KPs	General including KPs	KPs
Maturity of PrEP program in months	29	27	29	32	32	29	18

### Community Mobilization by Trained Enhanced Peer Mobilizers

For the non-governmental providers, community PrEP mobilization is conducted by a cadre of trained, part-time enhanced peer mobilizers (EPMs) representing lesbian, gay, bisexual, transgender and intersex (LGBTI) people and female SWs [22]. The EPMs, who work under a KP officer whose role is to provide support when required, are responsible for recruiting and linking clients to HIV testing services (HTS) during community outreach events. These activities are conducted outside normal working hours, typically at night, in one-on-one or small-group interpersonal communication sessions covering the benefits of testing, PrEP and ART use. Identified HIV-positive people are linked to ART services for care and treatment, while those who are HIV negative are referred to PrEP services. EPMs are paid a monthly $40 stipend. They also earn a pay-for-performance incentive of $10 for each person they test and successfully link to ART or PrEP.

### Typical Client Flow at PrEP Services

The annual follow-up schedule involves a total of eight visits for the government SGBV clinic and seven for the PSI facilities (Supplemental Table A2) as clients receive test results during the initiation visit. At the PSI clinics, clients present at reception for registration, proceed to HTS for confirmatory testing, and then see a PrEP provider for assessment of HIV risk and PrEP readiness prior to initiation. A peer counselor provides continuation advice and support while the client awaits test results and collection of PrEP drugs from the onsite clinic pharmacy and on the same day. A 2-week follow-up visit is booked for collection of blood test results. In the government clinic, an SGBV-trained nurse provides all the information and confirms HIV status, completes a PrEP risk assessment form, takes blood for diagnostics (liver function tests, full blood count, syphilis, creatinine and hepatitis B) and initiates a client on the same day with a 1-month drug supply. Clients are booked to return for a one-week follow-up visit to receive their blood test results.

### Cost Data Collection

We estimated the full economic cost of initiating and continuously engaging clients on oral PrEP from the providers’ perspective. This economic cost analysis includes actual financial costs incurred by both the PSI and government PrEP sites plus any donated goods and services and volunteer time, representing the full value of the resources used for PrEP service provision [23, 24]. The latter were accounted for during activity-based costing and site observations [25]. From November 2018 through January 2019, we collated financial reports and the associated PrEP monitoring and evaluation data to estimate the annual resource use for the retrospective 12-month period.

### Cost Analysis

The costing methods combined top-down expenditures analysis with ingredients-based costing consistent with guidelines [26–28]. The full economic costs for PrEP services were classified into two major cost categories: capital (including start-up training) and recurrent costs. The start-up costs of the initial training were treated as capital costs, because programs were assumed to benefit over a longer period, typically three to four years. Costs were further categorized by input type. Capital costs (including building space, equipment and vehicles) were amortized at a 3% discount rate [23, 24]. Time allocation sheets were analyzed using a time and motion exercise involving both self-reports and observation of staff providing PrEP and other services [29, 30].

Recurrent costs were comprised of personnel (both direct and support), management and administration, PrEP drugs, laboratory, pharmacy and promotional supplies, HIV tests, demand creation, and vehicle and building operation and maintenance costs [27]. The PrEP-related laboratory tests included chemistry analysis (creatinine test) and serology tests (syphilis, hepatitis B and pregnancy, as well as drug resistance tests for those who seroconvert while on PrEP).

In addition to the full fixed costs, as described above, we also estimated the incremental variable costs associated with each continuation visit and by specific population group (men—all ages; women—25 years and older; AGYW—15 to 24 years). Total program variable costs were estimated for all inputs related to continuation visits, such as staffing, PrEP drugs, HIV tests, follow-up calls and home visits. This approach allows us to estimate annual cost per client on oral PrEP as a function:

1 $$\$ {\text{ }}per{\text{ }}client = {\text{ }}f\left( {\$ fixed{\text{ }}initiation{\text{ }} + {\text{ }}number{\text{ }}of{\text{ }}continuation{\text{ }}visits{\text{ }}*{\text{ }}\$ {\text{ }}continuation{\text{ }}visit} \right)$$

We estimate the number of people needing to be initiated to achieve a full year of receiving oral PrEP as:

2 $$Q_{{InitiatesNeeded}} = 12{\text{ }}months/average{\text{ }}continuation{\text{ }}duration{\text{ }}in{\text{ }}months$$

And the cost per person year ($pPY) receiving oral PrEP is estimated as a function of Q_*initiatesNeeded*_:

3 $$\$ pPY\,{\text{receiving oral PrEP}} = {\text{ }}\$ _{{Initiation}} *Q_{{InitiationsNeeded~}} + {\text{ }}\$ _{{Continuation{\text{ }}visit}} *Q_{{ContinuationVisits{\text{ }}pp}} *Q_{{InitiationsNeeded}}$$

This function estimates how the $pPY on PrEP decreases as the duration of PrEP use increases. Average costs per client on PrEP decrease because the high fixed initiation costs are spread over more months of benefits.

### Sensitivity Analysis

To assess the impact of varying assumptions on the average cost per person initiated and per person continuing PrEP at 3 and 6 months, we conducted one-way sensitivity analyses [28]. Amortization time frames (economic life-years) for the initial training were varied between 1 and 6 years (base case is 4 years) and for furniture and equipment by 5 to 7 years (base case is 3 years) to assess the impact of project duration. We also conducted analyses to assess the impact of various scenarios (personnel salary, discounting, annual throughput, price of PrEP drug and PrEP program, with or without incentive for initiation) in anticipation of future program scale-up. Best- and worst-case scenarios, with all parameters yielding lowest/highest average cost per person initiated and continuously engaged on PrEP at 3 and 6 months, were estimated.

### Ethics

The MoHCC of Zimbabwe granted permission to conduct the study. Ethical approvals were obtained from the Medical Research Council of Zimbabwe (June 20, 2018) and the London School of Hygiene and Tropical Medicine Ethics Committee (April 18, 2018).

## Results

### Program Outcomes

Table 1 shows site characteristics including clinic location and size in visits per year. From January through December 2018, 4617 clients initiated oral PrEP at PSI clinics including the partner run site, ranging from 351 clients in Site 3 to 1599 clients in Site 1 (Table 2). At 3 and 6 months, respectively, 2099 (45%, range 10% to 79% across clinic sites) and 1142 (25%, range 7% to 47%) clients continued on PrEP in the PSI facilities. The government SGBV clinic initiated 60 clients on PrEP, with 17 (28%) and five (8%) continuing at three and six months, respectively. There were an average of 2.7 visits per client.

**Table 2:** Full economic and unit costs per PrEP client initiated and continued to 3 and 6 months at 7 Zimbabwe sites offering PrEP (2018)

Input	Site 1	Site 2	Site 3	Site 4	Site 5	Site 6	Site 7	ALL	Percent contribution (%)
Capital costs									
Buildings and storage	$3930	$954	$3540	$1898	$6470	$1202	$673	$18,666	2%
Site level equipment	$4851	$3961	$1101	$2200	$1751	$535	$164	$14,565	1%
Vehicles	$10,152	$6016	$3659	$5025	$7972	$5859	–	$38,683	3%
Start-up training (EPM's)	$64	$563	$229	$63	$422	–	$94	$1435	0%
Start-up training (Provider's)	$979	$377	$603	$904	$603	$301	$115	$3888	0%
Total capital	$19,976	$11,871	$9132	$10,090	$17,218	$7897	$1047	$77,253	7%
Recurrent costs									0%
Management & administration	$6200	$8208	$5001	$5386	$8427	$948	–	$34,169	3%
Personnel—direct	$59,196	$9233	$10,416	$34,145	$58,369	$44,823	$287	$216,469	19%
Personnel—support	$32,746	$24,426	$20,588	$34,372	$30,157	$5683	$97	$148,068	13%
PrEP drug	$25,902	$5431	$4297	$24,998	$28,068	$19,567	$1302	$109,567	10%
Other pharmacy supplies	$823	–	$89	–	$5283	–	–	$6195	1%
Lab supplies	$8228	$2571	$1805	$3312	$4150	$3682	$334	$24,082	2%
Promotional supplies	$557	$194	$197	$443	$368	–	$569	$2329	0%
HIV tests	$21,488	$5364	$3924	$17,267	$17,749	$13,534	$680	$80,006	7%
Demand creation—sub-grants	$85,287	$35,304	$15,100	$29,839	$58,499	$88,370	–	$312,399	28%
Demand creation—EPM incentives	$21,377	$6496	$5606	$10,562	$13,551	–	$831	$58,422	5%
Vehicle operation, & maintenance	$10,828	$7076	$6948	$6728	$2039	$7548	–	$41,167	4%
Building operation, & maintenance	$22	$231	$303	$39	$103	$2627	–	$3326	0%
Total recurrent	$272,655	$104,534	$74,274	$167,091	$226,763	$186,782	$4100	$1,036,199	93%
TOTAL	$292,631	$116,405	$83,406	$177,181	$243,981	$194,679	$5147	$1,113,430	100%
# PrEP clients initiated: AGYW	328	167	93	226	291	530	6	1641	1641
# PrEP clients initiated: adult women	601	35	107	298	203	112	37	1393	1393
# PrEP clients initiated: all men	670	298	151	120	313	74	17	1643	1643
# PrEP clients initiated: all	1599	500	351	644	807	716	60	4677	
Cost per PrEP client initiated	$183	$233	$238	$275	$302	$272	$86	$238	
# PrEP clients continued to 3 months	412	51	58	510	574	494	17	2116	
Cost per PrEP client continued to 3 months	$710	$2282	$1438	$347	$425	$394	$303	$526	
# PrEP clients continued to 6 months	236	34	32	213	379	248	5	1147	
Cost per PrEP client continued to 6 months	$1240	$3424	$2606	$832	$644	$785	$1029	$971	

### Total Costs and Cost Composition

The total annual program costs were $1,113,430 across the seven sites, with recurrent costs contributing 93% ($1,036,199) of the cost (Table 2); personnel was one-third of the cost. For the PSI sites, demand creation including mobilizer costs contributed 27% to the cost (Supplemental Fig. A1), and PrEP drugs 9%. For the government SGBV clinic, the largest cost (25%) was the PrEP drug (Supplemental Fig. A2).

### Average Costs

**Fig. 1:**
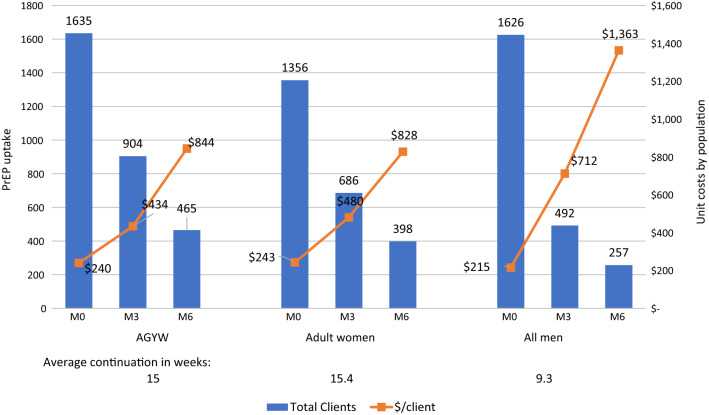
PrEP uptake (M0) and continuation at 3 and 6 months and average costs by population (PSI Zimbabwe only) (2018)

Table 2 shows average costs per person on PrEP for all seven sites and the breakdown of inputs. The average cost per person initiated on PrEP was $238, ranging from $183 at the largest PSI facility in Harare (Site 1) to $302 at the small clinic in a medium size town (Site 5) and $86 at the government SGBV clinic. Figure 1 shows average cost across populations, plotted against numbers initiated and continuing at months 3 and 6 across the six PSI sites. When assessed by population, the average costs per person initiated on PrEP across facilities appear similar, being lowest for all males (all ages) at $215, followed by $240 for AGYWs (ages 15 to 24), and highest for women (25 and older) at $243. Due to variable periods of engagement, average cost per client continuing on PrEP ranged from $347 to $2282 at three months and from $644 to $3424 at six months in the PSI sites. For the government SGBV clinic, the average cost per client continuing on PrEP at three and six months was $303 and $1029, respectively.

### Incremental Fixed and Variable Costs

We modeled the incremental costs of PrEP continuation visits, i.e., only those costs directly used, excluding any shared costs. Taking the full annual costs of PrEP provision minus the total incremental continuation costs generates the estimated full cost of initiation. To get the full cost per PrEP initiation and incremental cost per PrEP continuation visit, we divided the total costs by their respective outputs, i.e., initiations and continuation visits, as shown in Table 3. Across the clinics, the fully loaded fixed cost of a PrEP initiation visit was $178, and the incremental cost per follow-up PrEP visit was $22.

**Table 3:** Average cost per continuation visit and per person initiated excluding continuation visit costs at 7 Zimbabwe sites offering PrEP (2018)

	Site 1	Site 2	Site 3	Site 4	Site 5	Site 6	Site 7	All Sites
Total PrEP delivery costs	$292,631	$116,405	$83,406	$177,181	$243,981	$194,679	$5147	$1,113,430
Incremental cost of continuation visits								
ART/PrEP clinician	$9858	$1878	$1446	$11,483	$12,691	$9199	$374	$46,930
Site doctor	$58	$35	$26	$181	$222	$239	$0	$760
Pharmacy technician	$12,675	$2415	$1859	$14,764	$16,317	$11,827	$374	$60,231
PrEP peers & champions: allowances & home visits	$5400	$3240	$2700	$2160	$7425			$20,925
PrEP drugs	$19,116	$3267	$2786	$22,496	$24,881	$16,655	$1053	$90,252
HIV tests (weeks 4, 12, 28, 36, 48, 60, 72, 84)	$11,606	$2274	$1755	$13,287	$12,762	$9109	$309	$51,102
Phone charges	$5,328	$1,066	$888	$1,066	$1,066	$1,066		$10,478
Total Incremental continuation visit costs	$64,041	$14,175	$11,460	$65,437	$75,364	$48,093	$2110	$280,679
Full cost initiation^a^	$228,590	$102,230	$71,946	$111,744	$168,617	$146,586	$3036	$832,751
# of initiation visits	1599	500	351	644	807	716	60	4677
Full cost per initiation visit	$143	$204	$205	$174	$209	$205	$51	$178
# of continuation visits	2645	504	388	3081	3405	2468	151	12,642
Incremental cost per continuation visit	$24	$28	$30	$21	$22	$19	$14	$22
Average # continuation visits per client	1.7	1.0	1.1	4.8	4.2	3.4	2.5	2.7
Average duration per client in months	1.8	0.9	0.7	4.6	5.6	3.7	n/a	3.0
Average cost per person year	$1208	$3037	$4025	$710	$642	$876	n/a	$943

^a^Incremental cost per continuation visit = Total incremental continuation cost/#continuation visits; Full cost of initiation visits = Total PrEP delivery costs—Total incremental continuation costs; Full cost per initiation visit = Full cost of initiation visits/ #initiation visits; Average cost per person year: Eq. 2, e.g. site 1: $143*(12/1.8)

Using these fixed and variable costs per person, we can model the $pPY for different durations of PrEP continuation. We first estimated the number of people who need to be initiated to achieve 12 months of receiving oral PrEP following Eq. 2. To estimate the $pPY of receiving oral PrEP, commonly used in impact models, we applied Eq. 3. For example, a person-year on PrEP could be one person who continues for a year or 12 people who continue for a month. An estimate of the cost of the former would add the cost of one initiation visit and seven follow-up visits, while the latter would incur the cost of 12 initiation visits and 12 follow-up visits (see the annual visit schedule in Supplemental Table A2). The average duration of continuation was 13.1 weeks overall, generating an average $pPY cost of $943 ($642–$4025 across PSI clinics) (Table 3).

**Fig. 2:**
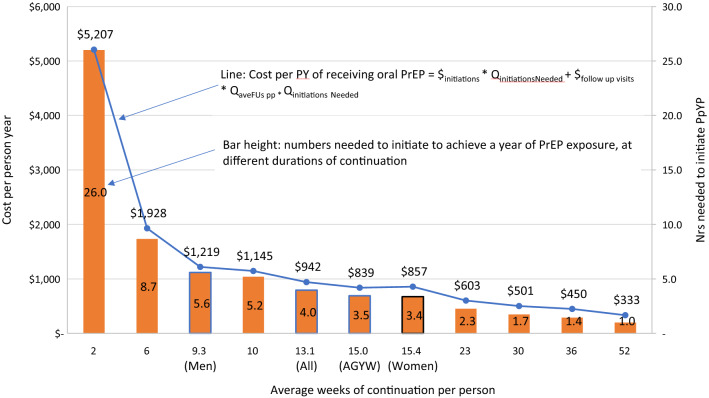
Cost per person-year and numbers needed to initiate by average continuation duration for Zimbabwe sites offering PrEP (2018)

By population, the average duration of continuation was 15.0 weeks for AGYW, 15.4 weeks for adult women and 9.3 weeks for all men. Figure 2 models out costs and numbers of clients needed to initiate across different average durations of continuation. For shorter continuation, more people need to be initiated to achieve a person-year receiving oral PrEP. For longer continuation, the fixed costs of initiation are spread over a longer time, reducing average $pPY of receiving oral PrEP. However, given differences in how long people continue on PrEP, $pPY shows more variability. While women of all age groups continued on PrEP for similar durations, resulting in very similar $pPY ($839 and $857, respectively among AGYW and adult women), men tended to discontinue PrEP sooner, with a consequent $pPY of $1,219.

### Sensitivity and Scenario Analysis

Supplemental Figure A3 depicts univariate and multivariate sensitivity and scenario analyses results for average costs per person initiated. Average costs remain largely robust under varying parameter specifications. Varying salary levels of PrEP providers 10% upwards and downwards resulted in respective average costs per client initiated of $229 and $246 (base case $238). When the price of PrEP drug was varied by 20% to assess the impact of price changes, average cost per client initiated was $236 and $240. Best- and worst-case scenarios, with all parameters yielding lowest/highest average cost per person initiated, resulted in average cost per client initiated of $222 and $249, respectively. Assessing costs without the EPM incentives resulted in average cost per client initiated of $225; however, this estimate assumes that uptake would not be affected. A full description of sensitivity analysis on the average cost per client continued at months three and six can be found in Supplement A1.

## Discussion

We estimated the annual total and unit economic costs of delivering oral PrEP to clients as part of integrated HIV and SRH services at seven Zimbabwe clinic sites. Total annual program costs were $1,113,430 and average costs for PSI $238, ranging from $183 at the largest facility in Harare (Site 1) to $302 at the smallest clinic (Site 5). Average costs appear lower at sites initiating larger numbers of PrEP clients, although an exception is the far lower initiation cost ($86) at the government SGBV site. Similar to other PrEP programs in sub-Saharan Africa costs in this study increase as clients discontinue PrEP [17–19].

Recent data from the POWER study [31] in Kenya and South Africa revealed that 25% (160/644) of AGYW restarted PrEP after a brief discontinuation [32]. This finding is consistent with findings of PrEP cycling in other regional programs, although few currently track PrEP restarts. Any restart at the sites in our study would have been captured as a costlier new PrEP initiation, making it difficult to estimate the true average cost per client if that client cycled on and off PrEP. Nevertheless, the finding of increased costs for sites with higher discontinuation rates, as seen in the PSI sites, merits further examination. One contributory factor could be the use of incentivized demand creation (via EPMs) for PrEP initiations, but not for follow-up and ongoing support of PrEP users.

Results show large variation across the sites, which influenced average costs. Varying lengths of PrEP program maturity help explain differences in average costs, because sites that began providing PrEP relatively earlier benefited from a longer period of program learning. Sites seeing more clients had lower average costs, highlighting potential economies of scale. Average costs were also influenced by type of staff employed, as sites employed staff with differing levels of specialization, and by how much staff were paid. Only three sites, for example, employed full-time doctors; the rest employed doctors for one day a week and for two to three hours on those days, depending on the volume of clients. It is therefore not surprising that the sites employing higher specialization cadres and with staff who were paid more tended to have higher costs highlighting potential cost savings if task shifting to lower, less paid cadres is implemented as shown by the government SGBV clinic.

Approaches to demand creation also influenced variation in average costs across sites; sites such as the government SGBV clinic and a smaller NGO clinic lacked funding for and therefore implemented few demand creation activities. The managers of those sites also expressed a fear of potentially creating unsustainable demand for PrEP in an environment of observed or anticipated drug stockouts.

Costs compare well with estimates elsewhere. A study in Kenya estimated the total and incremental cost of delivering PrEP integrated into a government health facility ART program, respectively, at $250 and $87 per couple per year [18]. Another study in Kenya found an average annual total cost of $602 for providing oral PrEP to one sex worker through the clinics of a sex worker outreach program (SWOP), including both direct and indirect costs [19], with personnel, PrEP drug and laboratory testing accounting for most of the cost differences compared to our findings. In our study laboratory testing costs constitute only 2% of costs because the programme funded laboratories in the PrEP sites perform this service at cost than would be the case if clients had to seek these services at private laboratories. Our initiation numbers suggest laboratory testing can restrict distribution mainly to sites that are large enough to have laboratories. Experience from elsewhere however has also shown higher costs of laboratory testing for creatinine and other tests to be a barrier to PrEP service delivery [10, 13, 33]. In Uganda, a study found that the annual cost of PrEP delivery for serodiscordant couples was $408 per couple in the study setting and $92 per couple in the government setting [17].

## Limitations

This study has several limitations. As noted, the sites included in the study did not track PrEP cycling, so any restarts would have been categorized as PrEP initiations, which have higher associated costs than restart or continuation visits. For this analysis, we deliberately selected seven sites that had already been providing PrEP for long enough to provide a year’s worth of cost data. A larger number of sites would provide more variability of average costs and greater insight into the cost drivers.

This average cost study is also limited to a provider perspective, excluding user costs incurred by clients seeking PrEP services. A costing study of the Jhpiego-led Jilinde project in Kenya includes the client as well as the provider costs of PrEP scale-up throughout Kenya, in preparation for national budgeting of future scale-up and to inform recommendations about which service delivery models are most effective at reaching specific populations [17, 18]. With more time and funding, this Zimbabwe PrEP study would have benefited from an exploration of the client costs of PrEP use and their potential as a contributor to not only uptake but also low continuation rates.

## Conclusions

Despite the above limitations, this costing study provides valuable information on the initiation and continuation costs of PrEP delivery services in Zimbabwe. A strength of this study is its ability to generate estimates of initiation and continuation costs based on actual program delivery experience. The analysis reveals ways by which costs can be reduced, most significantly through increased PrEP continuation. The findings of this costing study are both relevant and timely, as they come just as Zimbabwe begins to scale up PrEP delivery.

PrEP program outputs show high demand for PrEP across sites. However, challenges with continuation are evident and affect average costs, which increase rapidly as client continuation numbers drop off. As shown in this analysis a substantial proportion of PrEP clients drop off before the first month, some failing to return even for follow-up visit 1. This suggests that some people initiate and incur costs, though they either do not perceive themselves at risk or cannot overcome barriers to sustain PrEP use. Programs would benefit from economies of scale achieved through higher continuation rates albeit for those clients who continue to be at risk. Further research will help highlight what proportion of discontinuation is based on clients being no longer at risk, and what proportion is due to other reasons that could be managed, such as stigma, side effects, or the burden of regular pill-taking, including storage, consumption and knowledge of regimen [34]. It may also be important to assess the affordability and impact of extending the incentive for initiations to follow-up visits, in order to promote continued engagement where clients continue to be at risk. Such research will help frame strategies to improve continuation for those who still consider themselves at risk, to allow for natural cycling on and off PrEP when indicated together with better identification of clients more likely to continue use and those needing additional support. Tracking longitudinal client data to account for PrEP restarts will improve the accuracy of cost estimates and supporting clients at risk to continue on PrEP will improve program efficiency by spreading the fixed costs of initiation over more months of receiving oral PrEP.

## Supplementary Information

Below is the link to the electronic supplementary material.

Supplementary file1 (DOCX 48 kb)

Supplementary file2 (DOCX 51 kb)

Supplementary file3 (DOCX 52 kb)

Supplementary file4 (DOCX 52 kb)

Supplementary file5 (DOCX 49 kb)

Supplementary file6 (DOCX 46 kb)
