# Supplementary material for: Efficiency in PrEP Delivery: Estimating the Annual Costs of Oral PrEP in Zimbabwe
Source: AIDS Behav. 2021 Aug 27;26(1):161–70. doi: 10.1007/s10461-021-03367-w (PMC8786759; doi:10.1007/s10461-021-03367-w)
Supplement: Supplementary file 1 — Supplementary file1 (DOCX 48 kb) [file 10461_2021_3367_MOESM1_ESM.docx]

Table A1 Integrated services offered at 7 Zimbabwe sites offering PrEP (2018)

| **Facility Type** | **Facility (start date)** | **Scope of services provided** |
| --- | --- | --- |
| PSI clinic (6) | Site 1 (Nov 2016) | - Community peer mobilizers within routine demand creation activities - HIV testing services (HTS) - Family planning - Cervical cancer and STI screening & treatment - TB diagnosis & treatment, prevention - Post-rape counseling services - Viral load and other laboratory tests |
|  | Site 2 (Feb 2017) |  |
|  | Site 3 (Nov 2016) |  |
|  | Site 4 (Aug 2016) |  |
|  | Site 5 (Aug 2016) |  |
|  | Site 6 (Nov 2016) |  |
| Government clinic (1) | Site 7 (Jun 2017) | - Integrated into government SGBV services - Targets mostly key populations, e.g., FSWs, MSM and transgender populations - Subcontracted demand creation - HTS |
