# Supplementary material for: Efficiency in PrEP Delivery: Estimating the Annual Costs of Oral PrEP in Zimbabwe
Source: AIDS Behav. 2021 Aug 27;26(1):161–70. doi: 10.1007/s10461-021-03367-w (PMC8786759; doi:10.1007/s10461-021-03367-w)
Supplement: Supplementary file 2 — Supplementary file2 (DOCX 51 kb) [file 10461_2021_3367_MOESM2_ESM.docx]

Table A2 Annual PrEP visit schedule (baseline and follow-up [FU]) at 7 Zimbabwe sites offering PrEP (2018)

| **Months** | **Weeks** | **PSI** | **Government** |
| --- | --- | --- | --- |
| 0 | 0 | Baseline | Baseline |
|  | 1 |  | FU1 |
|  | 2 | FU1 |  |
|  | 3 |  | FU2 |
|  | 4 |  |  |
|  | 5 |  |  |
|  | 6 | FU2 |  |
|  | 7 |  | FU3 |
|  | 8 |  |  |
|  | 9 |  |  |
|  | 10 | FU3 |  |
|  | 11 |  |  |
|  | 12 |  | FU4 |
| 3 | 13 |  |  |
|  | 14 |  |  |
|  | 15 |  |  |
|  | 16 |  |  |
|  | 17 |  |  |
|  | 18 |  |  |
|  | 19 |  |  |
|  | 20 |  |  |
|  | 21 |  | FU5 |
|  | 22 |  |  |
|  | 23 | FU4 |  |
|  | 24 |  |  |
|  | 25 |  |  |
| 6 | 26 |  |  |
|  | 27 |  |  |
|  | 28 |  |  |
|  | 29 |  |  |
|  | 30 | FU5 | FU6 |
|  | 31 |  |  |
|  | 32 |  |  |
|  | 33 |  |  |
|  | 34 |  |  |
|  | 35 |  |  |
|  | 36 | FU6 |  |
|  | 37 |  |  |
|  | 38 |  |  |
| 9 | 39 |  | FU7 |
|  |  |  |  |
|  | 40 |  |  |
|  | 41 |  |  |
|  | 42 |  |  |
|  | 43 |  |  |
|  | 44 |  |  |
|  | 45 |  |  |
|  | 46 |  |  |
|  | 47 |  |  |
|  | 48 |  |  |
|  | 49 | FU7 |  |
|  | 50 |  |  |
|  | 51 |  |  |
| 12 | 52 |  | FU8 |
