# Supplementary material for: Efficiency in PrEP Delivery: Estimating the Annual Costs of Oral PrEP in Zimbabwe
Source: AIDS Behav. 2021 Aug 27;26(1):161–70. doi: 10.1007/s10461-021-03367-w (PMC8786759; doi:10.1007/s10461-021-03367-w)
Supplement: Supplementary file 3 — Supplementary file3 (DOCX 52 kb) [file 10461_2021_3367_MOESM3_ESM.docx]

Figure A1 Composition of costs in the 6 PSI Zimbabwe sites offering PrEP (2018)
