# Supplementary material for: Efficiency in PrEP Delivery: Estimating the Annual Costs of Oral PrEP in Zimbabwe
Source: AIDS Behav. 2021 Aug 27;26(1):161–70. doi: 10.1007/s10461-021-03367-w (PMC8786759; doi:10.1007/s10461-021-03367-w)
Supplement: Supplementary file 4 — Supplementary file4 (DOCX 52 kb) [file 10461_2021_3367_MOESM4_ESM.docx]

Figure A2 Composition of costs in the Zimbabwe government site offering PrEP (2018)
