# Supplementary material for: Efficiency in PrEP Delivery: Estimating the Annual Costs of Oral PrEP in Zimbabwe
Source: AIDS Behav. 2021 Aug 27;26(1):161–70. doi: 10.1007/s10461-021-03367-w (PMC8786759; doi:10.1007/s10461-021-03367-w)
Supplement: Supplementary file 5 — Supplementary file5 (DOCX 49 kb) [file 10461_2021_3367_MOESM5_ESM.docx]

**Figure A3 Sensitivity analysis: impact of assumptions on $/ client initiated for 7 Zimbabwe sites offering PrEP (2018)**
