# Supplementary material for: Efficiency in PrEP Delivery: Estimating the Annual Costs of Oral PrEP in Zimbabwe
Source: AIDS Behav. 2021 Aug 27;26(1):161–70. doi: 10.1007/s10461-021-03367-w (PMC8786759; doi:10.1007/s10461-021-03367-w)
Supplement: Supplementary file 6 — Supplementary file6 (DOCX 46 kb) [file 10461_2021_3367_MOESM6_ESM.docx]

Supplement A1. Sensitivity and scenario analysis for 7 Zimbabwe sites offering PrEP (2018)

The impact of varying assumptions on average cost per client continued are shown in supplemental Figure A3. Varying amortization time frames (economic life-years) for initial training between one and six years (base case is four years) across the seven sites resulted in average costs per client initiated of $238 and $241, costs per client continuing on oral PrEP at month three of $525 and $533 and costs per client continuing on oral PrEP at month six of $967 and $990. When amortization time frames for equipment were varied by 1 to 10 years (base case is 5 years) to assess the impact of longer or shorter project life, average costs per client initiated were $237 and $242, costs per client continuing on oral PrEP at month three were $523 and $537 and costs per client continuing on oral PrEP at month six were $967 and $990. When the discount rate was varied from 0% to 10% (base case is 3%) to assess the impact of either not discounting at all or a higher Reserve Bank of Zimbabwe rate, average costs per client initiated were $237 and $241, costs per client continuing on oral PrEP at month three were $524 and $532 and costs per client continuing on oral PrEP at month six were $967 and $981.

Varying personnel salary levels for PrEP providers 10% upwards or downwards resulted in average costs per client initiated of $229 and $246, costs per client continuing on oral PrEP at month three of $507 and $545 and costs per client continuing on oral PrEP at month six of $936 and $1,006. When the price of PrEP drug was varied 20% upwards and downwards to assess impact of price change or the availability of cheaper or more expensive drugs in the future, average costs per client initiated were $235 and $240, costs per client continuing on oral PrEP at month three were $521 and $531 and costs per client continuing on oral PrEP at month six were $961 and $980. A best and worst case scenario, with all parameters yielding lowest/highest average cost per person initiated and continuing on oral PrEP at three and six months, resulted in average costs per client initiated of $221 and $248, costs per client continuing on oral PrEP at month three of $491 and $550 and costs per client continuing on oral PrEP at month six of $905 and $1,015. Assessing costs without the incentive given to EPMs resulted in a average cost per client initiated of $225, a cost per client continuing on oral PrEP at month three of $499 and cost per a client continuing on oral PrEP at month six of $920.
